# Improving the Quality and Efficiency of Internal Referrals to Child and Adolescent Psychiatry: An Audit of Referral Standards in CAMHS

**DOI:** 10.1192/bjo.2025.10101

**Published:** 2025-06-20

**Authors:** Thuraya Obeid, Rhianne Edwards

**Affiliations:** Chester and Wirral Partnership, Chester, United Kingdom

## Abstract

**Aims:** Efficient and comprehensive referral processes are essential for the timely and appropriate delivery of psychiatric care within Child and Adolescent Mental Health Services (CAMHS). Incomplete or inconsistent referral documentation can lead to delays in care, increased administrative burden, and potential risks to patient safety. This audit evaluates the quality of internal referrals to CAMHS psychiatry, focusing on the inclusion of key demographic details, documentation of consent, evidence of prior therapeutic interventions, and risk assessment.

**Methods:** This retrospective audit examined referral quality within Marsden House CAMHS in Cheshire and Wirral Partnership NHS Foundation Trust (CWP). The focus was on internal referrals made by therapists and other CAMHS clinicians to the Psychiatry team. Data were collected from referral emails sent to the Marsden House Doctors’ email inbox between January and March 2024.The audit assessed 23 referrals against key quality indicators, including:Completeness of demographic details (name, age, gender, date of birth, NHS number).Documentation of patient consent within the referral.Evidence of prior therapy interventions (partnership work) before referral to psychiatry.Inclusion of risk assessment details.The number of emails exchanged per referral, indicating inefficiencies in the process.To ensure consistency in data extraction, a structured proforma was used to record key variables. The findings were then analysed to identify gaps in referral quality and inefficiencies in the process, with a focus on areas for improvement.

**Results:** The audit identified significant gaps in referral completeness, particularly in demographic documentation, consent recording, and risk assessment. The absence of a structured referral form contributes to inconsistencies and inefficiencies, increasing administrative workload and delaying access to psychiatric care. Additionally, a lack of documented prior therapy interventions suggests that referrals may not always align with NICE guidelines, which recommend therapy as the first-line treatment before psychiatric escalation.Key Findings: The mean number of demographic details included per referral was 2.7 out of 5 key identifiers. Consent was documented in only 43% (10/23) of referrals. 65% (15/23) of referrals documented prior therapy interventions, whereas 35% did not include this information. Risk was explicitly mentioned in only 39% (9/23) of referrals.On average, 2.43 emails were required per referral, highlighting inefficiencies in the process.The lack of a standardised referral form is a major contributing factor to these inefficiencies, leading to incomplete information, delays, and increased administrative workload. The findings also indicate that referrals do not always adhere to NICE guidelines, potentially leading to inappropriate psychiatric escalations when therapy should be the first-line intervention.

**Conclusion:** This audit highlights the need for a structured, standardised referral process to enhance efficiency, completeness, and patient safety in CAMHS psychiatry referrals. Implementing these recommendations will improve the quality of referrals, reduce delays, and ensure young people receive the most appropriate and timely psychiatric care.Recommendations: Implementation of a Standardised Referral Form with mandatory fields for demographics, consent, risk assessment, and prior therapy interventions.Training for Referrers to improve the quality and completeness of referrals in line with best clinical practice.Ensuring Compliance with NICE Guidelines by requiring documentation of prior therapy interventions before psychiatry referrals, unless clinically justified.Streamlining the referral process to improve efficiency and reduce unnecessary email correspondence.

